# Early bilateral and massive compromise of the frontal lobes

**DOI:** 10.1016/j.nicl.2018.02.026

**Published:** 2018-02-27

**Authors:** Agustín Ibáñez, Máximo Zimerman, Lucas Sedeño, Nicolas Lori, Melina Rapacioli, Juan F. Cardona, Diana M.A. Suarez, Eduar Herrera, Adolfo M. García, Facundo Manes

**Affiliations:** a Institute of Cognitive and Translational Neuroscience (INCyT), INECO Foundation, Favaloro University, Buenos Aires, Argentina; b National Scientific and Technical Research Council (CONICET), Buenos Aires, Argentina; c Universidad Autónoma del Caribe, Barranquilla, Colombia; d Center for Social and Cognitive Neuroscience (CSCN), School of Psychology, Universidad Adolfo Ibáñez, Santiago, Chile; e Centre of Excellence in Cognition and its Disorders, Australian Research Council (ACR), Sydney, Australia; f Laboratory of Neuroimaging and Neuroscience (LANEN), Institute of Translational and Cognitive Neuroscience (INCyT), INECO Foundation, Rosario, Argentina; g Instituto de Psicología, Universidad del Valle, Cali, Colombia; h Departamento de Estudios Psicológicos, Universidad ICESI, Cali, Colombia; i Faculty of Education, National University of Cuyo (UNCuyo), Mendoza, Argentina

**Keywords:** Frontal lobe, Neurodevelopmental disorders, Attention, Executive function, Language, Social cognition, Consciousness, DTI, MRI, fMRI

## Abstract

The frontal lobes are one of the most complex brain structures involved in both domain-general and specific functions. The goal of this work was to assess the anatomical and cognitive affectations from a unique case with massive bilateral frontal affectation. We report the case of GC, an eight-year old child with nearly complete affectation of bilateral frontal structures and spared temporal, parietal, occipital, and cerebellar regions. We performed behavioral, neuropsychological, and imaging (MRI, DTI, fMRI) evaluations. Neurological and neuropsychological examinations revealed a mixed pattern of affected (executive control/abstraction capacity) and considerably preserved (consciousness, language, memory, spatial orientation, and socio-emotional) functions. Both structural (DTI) and functional (fMRI) connectivity evidenced abnormal anterior connections of the amygdala and parietal networks. In addition, brain structural connectivity analysis revealed almost complete loss of frontal connections, with atypical temporo-posterior pathways. Similarly, functional connectivity showed an aberrant frontoparietal network and relative preservation of the posterior part of the default mode network and the visual network. We discuss this multilevel pattern of behavioral, structural, and functional connectivity results. With its unique pattern of compromised and preserved structures and functions, this exceptional case offers new constraints and challenges for neurocognitive theories.

## 1 Introduction

From classical behavioral frameworks (Mesulam, 1986) to current neurocognitive theories (Donaghy, 2007; Stuss and Levine, 2002; Torralva et al., 2016) and even recent network approaches (Braun et al., 2015; Hampshire and Sharp, 2015), all conceptions of brain function have highlighted the complexity of the frontal lobes. These structures constitute the chief executive component in a large hierarchy of control mechanisms (Fuster, 2001), playing critical roles in domain-general functions, such as executive control (Badre, 2008) and abstraction capacity (Badre and D'Esposito, 2009; Diamond, 2006). Moreover, they are key contributors to other specific functions, such as decision making (Rushworth et al., 2011), consciousness (Lau and Rosenthal, 2011), memory (Kurby and Zacks, 2008), language (Hage and Nieder, 2016), and social cognition (Amodio and Frith, 2006). Its structural connections, comprising 12 pathways along a rostrocaudal axis, provide links across the whole brain (Thiebaut de Schotten et al., 2016). Similarly, this region includes key hubs of multiple functional networks, including intrinsic frontal connections (Badre and D'Esposito, 2009; Diamond, 2006) and extended circuits, such as the cingulo-opercular (or salience) network, the default mode network (DMN), the fronto-striatal network, and the domain-general frontoparietal network (FPN) (Hampshire and Sharp, 2015).

While the above insights have been derived from multiple approaches, no study has yet profited from the unique opportunity to study the multidimensional impact of massive developmental affectation of frontal lobes. Indeed, while cases of cerebellar, temporal, and callosal agenesis have been repeatedly documented, there seems to be no report of this condition with preservation of temporo-posterior structures. Partial frontal compromise has been described in cases of temporal agenesis (Kansu and Zacks, 1979), frontal ependymal or arachnoid cysts (Sarnat and Flores-Sarnat, 2016), hydrocephalus (Feuillet et al., 2007), and early or adult-onset strokes (Payne and Lomber, 2001; Price et al., 1990). Moreover, although extended patterns of frontal insult have been observed in holoprosencephaly (e.g., Liasis et al., 2009), hydranencephaly (Segawa et al., 2007), and other neurodevelopmental conditions (Li et al., 2016; Sarnat and Flores-Sarnat, 2016), these are accompanied by massive damage to temporal and posterior regions. Thus, a case of nearly complete and selective bilateral frontal affectation represents an unprecedented source of insights into this structure's functional and plastic properties, with potential implications for neurocognitive modeling.

Here we report the case of GC, an eight-year-old girl with massive frontal affectation of unknown pathogenesis and molecular basis. The patient presents complete absence of the several frontal bilateral structures. However, temporal, parietal, occipital, and cerebellar regions seemed preserved.

## 2 Patient and methods

### 2.1 Case GC

**Fig. 1:**
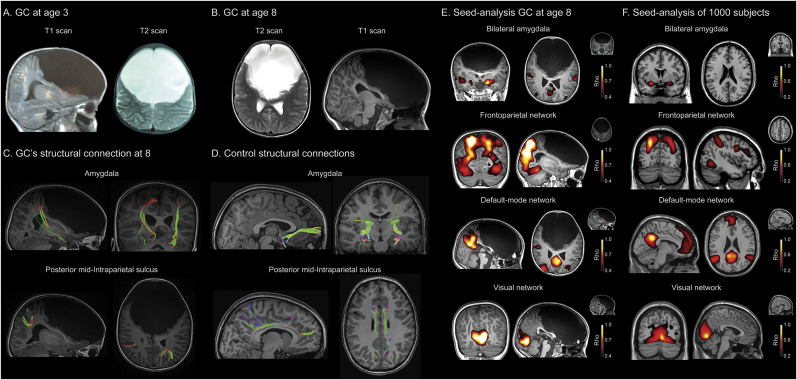
Imaging evidence for bilateral frontal compromise. A–B: Structural MRI. (A) GC's first report of frontal compromise at age three. MRI scans revealed no structures in the frontal lobe, covered with cerebrospinal fluid. Weighed-T1 MRI scans showed no recognizable frontal structures, expect for a small portion of the ventral frontal cortex. The mesencephalon, pons, and medulla oblongata were present, and so were all other lobes and the cerebellum. Cortical gyri were relatively preserved, as were the shape and proportion of the lateral, third, and fourth ventricles. (B) GC's report at age 8: T2 axial image. Original T2 and T1 sequences showing views of the patient's brain. Only a small portion of the ventral frontal cortex was evident, resembling a ventrolateral portion of the orbitofrontal cortex. For more views, see Fig. 2, and Supplementary Video 11. C–D: DTI. (C) Structural connections of GC at age 8: amygdala (top) and posterior mid-intraparietal sulcus (mid-IPS, bottom). (D) Structural connections of a healthy control matched with GC: amygdala (top) and mid-IPS (bottom). A comparison with DTI trajectories from a healthy control revealed amygdalar temporo-posterior network preservation and atypical anterior connectivity, alongside pervasive changes in the spatial and directional spread of mid-IPS fibers (intraparietal sulcus). Coloring of the white matter fibers is based on the following conventions: red: medial-lateral; green: anterior-posterior; blue: inferior- superior. E–F: Seed-analysis. (E) Seed-analysis of GC's resting-state fMRI recordings at age 8. Correlation maps were thresholded at Z > 0.04 (to show the strongest associations) of the bilateral amygdalar, frontoparietal, default-mode, and visual networks. (F) Seed-analysis of 1000 subjects. Correlation maps were thresholded at Z > 0.02 (to show the strongest associations) of the bilateral amygdalar (MNI seed-voxel coordinates, x = −26, y = 2, z = −16, and x = 22, y = −6, z = 12), frontoparietal (MNI seed-voxel coordinates, x = −23, y = −70, z = 46), default-mode (MNI seed-voxel coordinates, x = −12, y = −50, z = 32), and visual (MNI seed-voxel coordinates, x = 6, y = −78, z = −3) network. Cyan dots indicate seed location for the analysis of each network. All images are shown in neurological orientation. (For interpretation of the references to color in this figure legend, the reader is referred to the web version of this article.) Imaging evidence for bilateral frontal compromise. A–B: Structural MRI. (A) GC's first report of frontal compromise at age three. MRI scans revealed no structures in the frontal lobe, covered with cerebrospinal fluid. Weighed-T1 MRI scans showed no recognizable frontal structures, expect for a small portion of the ventral frontal cortex. The mesencephalon, pons, and medulla oblongata were present, and so were all other lobes and the cerebellum. Cortical gyri were relatively preserved, as were the shape and proportion of the lateral, third, and fourth ventricles. (B) GC's report at age 8: T2 axial image. Original T2 and T1 sequences showing views of the patient's brain. Only a small portion of the ventral frontal cortex was evident, resembling a ventrolateral portion of the orbitofrontal cortex. For more views, see Fig. 2, and Supplementary Video 11. C–D: DTI. (C) Structural connections of GC at age 8: amygdala (top) and posterior mid-intraparietal sulcus (mid-IPS, bottom). (D) Structural connections of a healthy control matched with GC: amygdala (top) and mid-IPS (bottom). A comparison with DTI trajectories from a healthy control revealed amygdalar temporo-posterior network preservation and atypical anterior connectivity, alongside pervasive changes in the spatial and directional spread of mid-IPS fibers (intraparietal sulcus). Coloring of the white matter fibers is based on the following conventions: red: medial-lateral; green: anterior-posterior; blue: inferior- superior. E–F: Seed-analysis. (E) Seed-analysis of GC's resting-state fMRI recordings at age 8. Correlation maps were thresholded at Z > 0.04 (to show the strongest associations) of the bilateral amygdalar, frontoparietal, default-mode, and visual networks. (F) Seed-analysis of 1000 subjects. Correlation maps were thresholded at Z > 0.02 (to show the strongest associations) of the bilateral amygdalar (MNI seed-voxel coordinates, x = −26, y = 2, z = −16, and x = 22, y = −6, z = 12), frontoparietal (MNI seed-voxel coordinates, x = −23, y = −70, z = 46), default-mode (MNI seed-voxel coordinates, x = −12, y = −50, z = 32), and visual (MNI seed-voxel coordinates, x = 6, y = −78, z = −3) network. Cyan dots indicate seed location for the analysis of each network. All images are shown in neurological orientation. (For interpretation of the references to color in this figure legend, the reader is referred to the web version of this article.)

GC was a firstborn delivered at 40 weeks following a Cesarean section for footling breech presentation. There were no prenatal complications due to infections, trauma, drug abuse, or any other chronic disease. At birth, she was 52 cm tall and weighed 3000 g. Her mother and grandmother suffered from depression and schizophrenia, respectively, but there were no familial antecedents of neurological conditions or brain malformations. GC exhibited cephalic support at 5 months and achieved a stable sitting position at 9 months. Fontanelle closure was slightly delayed. At 6 months she exhibited symptoms of probable developmental disorder and was diagnosed with presumed perinatal hypoxia (although diagnosis was later nullified). She uttered her first words at 18 months, began walking at 23 months, and developed structured language when she turned 3. At this age, after presenting motor symptomatology and the first signals of disinhibition and impulsivity, she underwent her first MRI scanning (Fig. 1A–B), which showed that the anterior fossa was almost completely filled by cerebrospinal fluid. Accompanying neuropsychological assessments at this stage revealed a low IQ, disinhibition, and impairments of memory, language, and attention. However, she successfully attended a regular kindergarden from ages 3 through 5. In 2016, having turned 5, she began primary school but was expelled three months later due to impulsive behavior and recurrent aggression to her peers. Ever since, CG's behavior has been characterized by irritability, disruption of social norms, and impulsivity. External (physical and familial) assistance is constantly required to organize her behaviors. She has received occupational therapy, language therapy, and physiotherapy, but only sporadically. The reported evaluation was done at the patient's age 8 (see Supplement 2 for a detailed description). All participants (patient's parents, as well as controls, see below) provided written informed consent in agreement with the Declaration of Helsinki, and the study was approved by the Ethics Committee of the Institute of Cognitive Neurology (INECO).

### 2.2 Neuropsychological assessment

We used a systematized battery of neuropsychological functions (see Supplement 1) to evaluate attention (visual and auditory attention); memory encoding, language, praxis, and emotional processing. All behavioral tests took place at the clinical center over two consecutive days. The evaluations were performed in 20/30-minute periods, with breaks initiated by either the examiner or the patient. Some subtests could not be administered due to GC's refusal to complete them. Given that the patient refused to complete some subtests, insights into these domains were gained through a clinical interview for frontal disorders (Mesulam, 2000). Some of these interactions were recorded and edited in short videos (Supplementary videos).

### 2.3 Imaging recordings and analysis (MRI, DTI, fMRI)

#### 2.3.1 MRI

The first MRI recordings of GC are from the year 2011, when she was three years old. In this session, sagittal and axial T1 and T2 images were acquired with a 3 T Siemens scanner. In the year 2016, at the age of eight, GC underwent another scanning session in a 3 T Siemens scanner with a standard head coil. Structural T1 scans were acquired with the following parameters: matrix size = 247 × 206 × 213, 1 mm isotropic, TR = 2200 ms, TE = 2000 ms and flip angle = 90. Axial T2 sequences were also obtained.

#### 2.3.2 DTI

We implemented a HARDI scheme, with the following parameters: (i) a total of 128 diffusion sampling directions, (ii) b-value = 1000 s/mm^2^, (iii) in-plane resolution of 0.647059 mm, and (iv) slice thickness of 5.85 mm. To show the normal patterns of white matter tracks, we obtained data from a control subject (an eight—year-old, right-handed female) from the Pediatric Imaging, Neurocognition, and Genetics (PING) project (http://pingstudy.ucsd.edu/), downloaded through the NITRC portal (https://www.nitrc.org/). For this control subject, diffusion images were acquired on a GE SIGNA HDx scanner using a diffusion sequence (PING_PROTOCOL_01_21_10/4), with TE = 83 ms, and TR = 13,600 ms. A DTI diffusion scheme was used, and a total of 30 diffusion sampling directions were acquired. The b-value was 1000 s/mm^2^. The in-plane resolution was 1.875 mm. Slice thickness was 2.5 mm.

#### 2.3.3 fMRI

As in previous works of single subject fMRI recordings (Garcia et al., 2017), the protocol lasted 9 min and 180 volumes were obtained. GC was sedated during the procedure given her difficulties to stay as still as possible. Several studies have shown that even under sedation, resting-state networks are still partially preserved and can be correctly identified [for a review, see (Heine et al., 2012)]. In particular, the DMN, FPN, and visual network are still preserved and can be identified under light/moderate conditions (Boveroux et al., 2010; Greicius et al., 2008; Martuzzi et al., 2010; Stamatakis et al., 2010), and even unconsciousness level of sedation (Boveroux et al., 2010; Martuzzi et al., 2010).

Functional networks for the control sample were extracted from Neurosynth (http://www.neurosynth.org/), a validated on-line platform (Yarkoni et al., 2011) that automatically synthesizes results from over 11,000 neuroimaging studies. Results from this database have been used in previous functional connectivity research (Kong et al., 2017; Lieberman and Eisenberger, 2015; Pauli et al., 2016). The correlation maps for the bilateral amygdala, FPN, DMN, and visual network were based on resting-state functional connectivity analysis on 1000 subjects, provided to Neurosynth courtesy of Thomas Yeo, Randy Buckner, and the Brain Genomics Superstruct Project (https://dataverse.harvard.edu/dataverse/GSP) [for details regarding acquisition, preprocessing, and analysis, see (Buckner et al., 2011; Choi et al., 2012; Yeo et al., 2011)]. Seeds used to estimate these networks were selected from Fox et al. (2005) (MNI coordinates, x = −23, y = −70, z = 46) for the FPN, from Williams et al. (2006) for the bilateral amygdala (MNI coordinates, x = −26, y = 2, z = −16, and x = 22, y = −6, z = −12), from Greicius et al. (2003) for the DMN (MNI coordinates, x = −12, y = −50, z = 32), and from De Luca et al. (2006) for the visual network (MNI coordinates, x = 6, y = −78, z = −3). Their coordinates were introduced in the platform to generate seed-voxels to obtain a correlation map for each network comprising all co-activated brain regions across the resting-state fMRI time series of the 1000 subjects. Then, each correlation map was downloaded from the platform and overlapped in a MNI-T1 template with a threshold of Z > 0.02 to show only the strongest associations of each network.

#### 2.3.4 DTI preprocessing

Note that in previous single case reports of agenesis or massive compromise assessed with DTI (e.g., Ronconi et al., 2017; Yu et al., 2015), only the patient's DTI data was provided given that canonical tracts present standard pathways, despite small differences. In this work we additionally included a single case to illustrate the canonical normal structural connections and to evidence how preserved or disrupted the patient's structural connections are.

As in other single cases of brain agenesis or major brain compromise (e.g., Ronconi et al., 2017; Yu et al., 2015), anatomical MRI data was co-registered and re-sliced into the diffusion MRI data using SPM. The co-registration of the anatomical MRI to the DTI data was not challenging as both the anatomical MRI and the diffusion MRI data share the absence of frontal lobes. A diffusion tensor was calculated after the diffusion MRI data was slice-orientation-corrected to the b-table. The slice orientation correction to the b-table depends exclusively on the orientation of the slice and on the form of the b-table, and it is not dependent on the shape of the brain. Thus, the absence of frontal lobes causes no problems for this correction. Anatomical MRI data was co-registered and re-sliced into the diffusion MRI data using SPM, and the diffusion MRI data was processed using DSI Studio (http://dsi-studio.labsolver.org). White matter fiber tracks were obtained by using pairs of anatomically-defined regions, where one defines a group of track-generation seed points (TSP), and the other acts as a Region-of-Interest (ROI) which the tracts need to touch for them to be preserved. For some white matter fibers, we also used Region-of-Avoidance (ROA) analyses, specifying areas which fibers cannot touch (see Basser et al., 2000; Conturo et al., 1999; Lori et al., 2002). The same procedure was implemented for GC and the control, the only difference being the location of the TSP, ROI, and ROA, given the particularities of GC's brain anatomy.

#### 2.3.5 FMRI preprocessing

Preprocessing and resting-state network estimations were implemented with the DPARSF toolbox (Chao-Gan and Yu-Feng, 2010), as in previous works of our group (Abrevaya et al., 2017; Garcia-Cordero et al., 2016; Melloni et al., 2016; Sedeno et al., 2016; Sedeno et al., 2014; Sedeno et al., 2017; Yoris et al., 2017). The first functional images were discarded; the rest were slice-time corrected, realigned to the middle slice of the volume, band-pass filtered (0.01–0.08 Hz), and finally smoothed with an 8-mm full-width half-maximum kernel. GC showed movements no >1.5 mm (right = 0.01 mm; forward = 0.04 mm and up = 0.02), and/or rotations higher than 1.5° (pitch = 0.01°; roll = 0.01°; yaw < 0.01°). However, to remove potential variance introduced by spurious sources, we also regressed out the six movement parameters, along with the average signal of the ventricular CSF and white matter (Van Dijk et al., 2012). Given that all the analyses were performed on GC's native space, images were not normalized to any standard template – to avoid deformations due to this transformation process.

For the analyses of the bilateral amygdala, the DMN, the FPN, and the visual network, we located 5-mm-diamter spheres as seeds in the left and right amygdalae (Williams et al., 2006), in the left posterior cingulate cortex (Greicius et al., 2003), the left-intraparietal sulcus (Fox et al., 2005), and the right lingual gyrus (De Luca et al., 2006), respectively. As our analysis was performed on GC's native space, the seeds' positions in these areas were determined by an expert neurologist [FM]. Then, BOLD signal time-courses were extracted from the voxels within each seed region and correlated to every other voxel in the brain using Pearson's correlation coefficient. Next, a Fisher z-transformation r-to-z was performed.

## 3 Results

### 3.1 Neurological and neuropsychological assessment

Poor control and regulation of behaviors were the hallmark of GC's deficits (frontal disinhibition syndrome). No signs of frontal abulic syndrome were evident. She could describe sensory and affective experiences, and reacted to environmental events with apparent emotional and cognitive congruency (e.g., pleasure, tiredness, playfulness, anger, and basic symbolization; Supplementary Video 1, Supplementary Video 2). Her basic motor repertoire was characterized by perseveration, but muscle bulk, muscle tone, posture, and strength were normal. Bilateral mirror movements were observed alongside affected rapid alternating movements. There was partial dysmetria on finger-to-nose (Supplementary Video 3) and heel-knee-shin tasks.

Structured neuropsychological tests revealed strong executive control and abstraction deficits, accompanied by partial preservation of other domains including language and communication, memory, spatial cognition and socioemotional behaviors (Table 1, Supplementary Video 1, Supplementary Video 2, Supplementary Video 3, Supplementary Video 4, Supplementary Video 8, Supplementary Video 9, Supplementary Video 10, Supplement 2).

### 3.2 Imaging

**Fig. 2:**
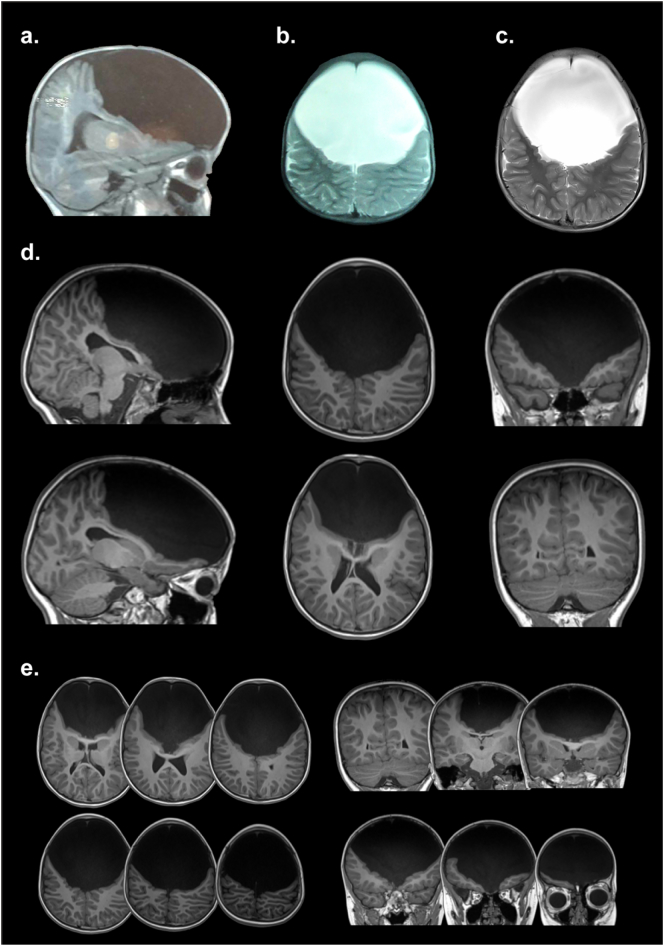
Detailed Structural results. (a–b) GC's first report at age three. MRI scans revealed no structures in the frontal lobe, covered with CFS. Weighed-T1 MRI scans showed no recognizable frontal structures, expect for a small portion of the ventral frontal cortex. The mesencephalon, pons, and medulla oblongata were present, and so were all other lobes and the cerebellum. Cortical gyri were relatively preserved, as were the shape and proportion of the lateral, third, and fourth ventricles. (c) GC's report at age 8: T2 axial image. (d) Structural images at age 8: Original T1 sequence showing sagittal, axial, and coronal views of the patient's brain. (e) Multislice images at age 8: axial (from ventral to dorsal slices) and coronal (from posterior to anterior) views. Only a small portion of the ventral frontal cortex was evident, resembling a ventrolateral portion of the orbitofrontal cortex. All images (a to e) are shown in neurological orientation. For more views, see Supplementary Video 11. Detailed Structural results. (a–b) GC's first report at age three. MRI scans revealed no structures in the frontal lobe, covered with CFS. Weighed-T1 MRI scans showed no recognizable frontal structures, expect for a small portion of the ventral frontal cortex. The mesencephalon, pons, and medulla oblongata were present, and so were all other lobes and the cerebellum. Cortical gyri were relatively preserved, as were the shape and proportion of the lateral, third, and fourth ventricles. (c) GC's report at age 8: T2 axial image. (d) Structural images at age 8: Original T1 sequence showing sagittal, axial, and coronal views of the patient's brain. (e) Multislice images at age 8: axial (from ventral to dorsal slices) and coronal (from posterior to anterior) views. Only a small portion of the ventral frontal cortex was evident, resembling a ventrolateral portion of the orbitofrontal cortex. All images (a to e) are shown in neurological orientation. For more views, see Supplementary Video 11.

A 3 T MRI scan revealed almost complete absence of the frontal lobe (with a large extraparenchymal cyst filled with CSF in the whole anterior fossa). Only a minor portion of the ventral territory seemed preserved (Figs. 1B and 2, Supplementary Video 11). Temporal, parietal, occipital, and cerebellar structures, as well as mesencephalon, pons, and medulla oblongata were present and apparently normal in spite of some expected compression.

**Fig. 3:**
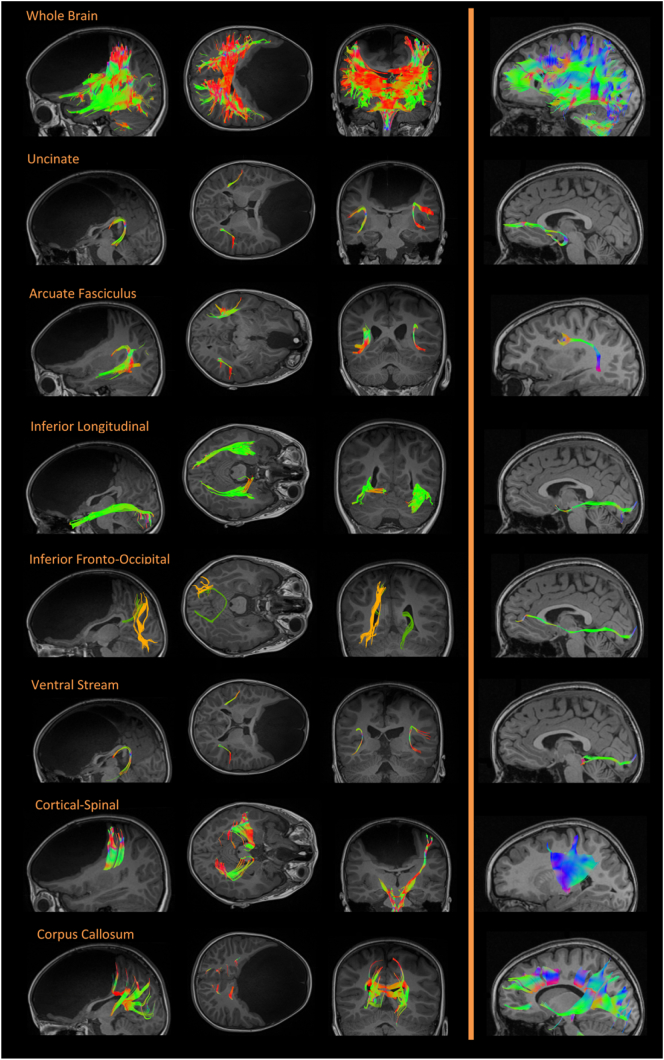
Additional structural connections. The first three columns show DTI results of the patient and the right-side column shows results from a matched healthy control. Rows correspond to different white matter connections, namely: whole brain, uncinate fasciculus, arcuate fasciculus, inferior longitudinal fasciculus, inferior fronto-occipital fascicle, ventral stream, cortical-spinal tract, and corpus callosum. DTI shows the color-coded ascending and descending fiber, as well as the anterior-posterior fibers. A comparison with DTI trajectories from a healthy control revealed pervasive changes in the spatial and directional spread of fibers. Coloring of the white matter fibers is based on the following color code: red: medial-lateral; green: anterior-posterior; blue: inferior-superior. (For interpretation of the references to color in this figure legend, the reader is referred to the web version of this article.)

We investigated the structural connections (DTI) of the amygdala and the mid-intraparietal sulcus (mid-IPS, Fig. 1C, top & bottom). Both areas normally have prefrontal connectivity. A qualitative comparison from an age- and gender-matched healthy control (Figs. 1D, 3) with Fractional anisotropy showed almost complete lack of frontal fibers between the amygdala and frontal regions (Fig. 1C, top). Only a small and atypical group of fibers were preserved in the frontal ventral region. In the patient, atypical connections were observed in the amygdala and the cuneus (Fig. 1C). Regarding the mid-IPS, the patient exhibited abnormal tracts connecting posterior, occipital and even cerebellar regions (Fig. 1C, bottom), in comparison with the classical fronto-parietal connections observed in the healthy control (Fig. 1F). Complementary analyses showed multiple absent tracts in the frontal regions (Fig. 3).

**Table 1:** Neuropsychological performance of the patient.

Cognitive domain	Task	Performance (%)	Patient's responses
Attention and control	Cancellation	0	N
	Visual letter cancellation	0	N
	Digits forward	25	Y
	Digits backward	25	Y
Verbal and visual memory coding	Word learning	39	Y
	Geometrical figure learning	0	N
Delayed verbal recall	Free recall	33.3	Y
	Cue recall	55.5	Y
	Recognition	66.5	Y
Delayed visual recall	Free recall	0	N
	Cue recall	0	N
	Visual recognition	0	N
Language	Syllables repetition	12.5	Y
	Words repetition	62.5	Y
	Non-words repetition	25	Y
	Sentences repetition	37.5	Y
	Objects naming	60	Y
	Pointing	100	Y
	Discourse comprehension	65	Y
Praxis	Complex figure (copying)	0	N
Visual perception	Superimposed figures	50	Y
	Spatial orientation	12.5	Y
	Line orientation	0	N
Emotions	Fear	40	Y
	Disgust	60	Y
	Anger	60	Y
	Surprise	20	Y
	Sadness	40	Y
	Happiness	100	Y

Percentage of correct responses in each task. The rightmost column indicates whether the patient provided responses (task engagement) for each task. Y: yes. N: no.

We explored two fMRI functional connectivity seeds on amygdala and the mid-IPS, the posterior part of the frontoparietal network (FPN). Results were compared with data from the Connectome project. In comparison with controls (Fig. 1E–F), GC evinced an almost complete lack of connections between the amygdala and frontal regions, together with atypical connections between the amygdala and the territory of the DMN (cuneus, Fig. 1E). FPN connectivity showed abnormal connections of posterior, occipital, and even cerebellar regions in the patient (Fig. 1E), relative to the classical fronto-parietal connections observed in the healthy controls (Fig. 1F). As a complementary analysis, we measured the DMN and the VN (Fig. 1E–F). Both networks were unexpectedly preserved, with the DMN resembling the posterior cingulate-precuneus territory (although no anterior part of the network was present) and the VN displaying the expected spatial extension (Fig. 1E).

## 4 Discussion

To our knowledge, this is the first case of nearly complete and selective neurodevelopmental affectation of the frontal lobes, presenting with marked deficits in abstraction, attention, and cognitive control. She exhibited partial preservation of sensorimotor (walking and sensory abilities) and cognitive (consciousness, language, memory, social interaction) functions. Though deficits in these domains were present, they were much less pervasive than would be expected. Neurodevelopmental disorders are crucial to identify critical neurocognitive functions that resist neurodevelopmental adaptation (Kansu and Zacks, 1979; Ronconi et al., 2017; Yu et al., 2015). This case suggests that, despite probable developmental and neuroplastic changes, the absence of frontal lobes inexorably impairs its classical putative functions, namely, executive control and abstraction (Badre, 2008; Badre and D'Esposito, 2009; Diamond, 2006; Mesulam, 1986), as well as contextual appropriateness and behavioral self-regulation (Baez et al., 2017; Baez et al., 2016; Burgess et al., 2009; Ibanez et al., 2017; Ibanez and Manes, 2012; Melloni et al., 2016). Conversely, a set of sensorimotor (basic walking and sensory abilities) and cognitive (basic levels of consciousness, language, memory, social interaction) functions were partially spared. Among the affected frontal areas, the anterior insula is also involved in some of the preserved functions, such as a consciousness, social cognition, interoception, and emotion processing (Adolfi et al., 2017; Baez et al., 2014; Couto et al., 2015; Couto et al., 2013; Garcia-Cordero et al., 2017; Ibanez et al., 2010; Ibanez et al., 2013; Melloni et al., 2014; Santamaria-Garcia et al., 2017; Vicario et al., 2017; Yoris et al., 2017). Though deficits in these domains were clearly present, they were much less pervasive than would be expected in the face of fronto-insular underdevelopment.

The preservation of motor activity and motor cognition may be explained by plasticity and compensation via other motor structures, such as the basal ganglia and the cerebellum (Leisman et al., 2014). Instead, subtler impairments seem to reflect the absence of prefrontal structures and their subcortical connections (Bostan et al., 2013). Also, the sparing of basic sensory and perceptual domains may reflect the integrity of the postero-temporal ventral perceptual stream and the partial preservation of the ventral-lateral prefrontal cortex and its connections (Faw, 2003).

Similarly, this case offers an alternative model for the classical ventral and dorsal accounts of apraxia and agnosia. Here, the overall pattern of preserved basic functions and affected complex functions may be related to the absence of frontal mechanisms and related working memory deficits (Pisella et al., 2006), with preserved parietal and posterior regions specialized in lower-level operations. Similarly, deficits in gnosis and general praxis, together with limb-kinetic apraxia and apraxia of speech, may reflect the partial reliance of these domains on frontal structures (Acciarresi, 2012).

GC also demonstrated preserved conscious functions. She showed well-defined wakefulness states, with several sensory and emotional experiences, including explicit self-other distinctions. This suggests that pre-attentive (primary or phenomenal consciousness) and more elaborate forms of consciousness were unaffected. This case supports previous evidence of preserved conscious states in hydranencephalic children (Merker, 2007), ongoing frontal removal (Penfield and Jasper, 1954), and other conditions (Merker, 2007), undermining the recently reedited role (Lau and Rosenthal, 2011) of the frontal lobes in consciousness. Arguably, compensatory or sufficient mechanisms would comprise the brainstem (Merker, 2007) and posterior regions (Koch et al., 2016).

GC's long-term memory remained partially functional. Deficits may reflect reentrant loops of fronto-posterior structures (Eriksson et al., 2015) and related functions of attention and working memory (Kurby and Zacks, 2008), whereas preserved patterns may depend on the temporal cortex and other posterior structures (Kurby and Zacks, 2008). Similarly, GC retains basic language and communicative skills. Especially noteworthy are her spared speech production skills despite the apparent absence of bilateral Broca's areas and related motor cortices (to our knowledge, this is the first report of such a pattern). The most preserved domains (word repetition, word/non-word dissociation, naming) seem to depend on the temporal stream (Leonard and Chang, 2014), which was considerably unaffected. Also, GC' exhibited spared (though reduced) communicative intention. This suggests that ventrolateral prefrontal language networks in the language-dominant hemisphere (Faw, 2003) are not critical (in the presence of neurodevelopmental changes) when the temporo-posterior language stream is uncompromised.

In addition, the frontal lobes seem to be implicated in social and emotional processes (Amodio and Frith, 2006; Ibanez et al., 2018; Ibanez et al., 2014; Ibanez and Manes, 2012; Ibáñez et al., 2017; Stanley and Adolphs, 2013). However, the patient presented some normal social cognition and emotions. Of course, beyond these basic skills, explicit, reflective, and high-level social cognition (i.e., second-order theory of mind) was impaired. Social cognition and emotions seem to intensely depend on distributed mechanisms and networks, with frontal lobes supporting high-level processing (Amodio and Frith, 2006; Ibanez and Manes, 2012; Stanley and Adolphs, 2013). Conversely, temporal, parietal, and subcortical structures (including the basal ganglia, the amygdala, and the cerebellum) are also engaged in different aspects of social and emotional process. The posterior DMN has been related to social cognition (for a review, see Li et al., 2014) and this network was functionally preserved in GC. In brief, the patient's socioemotional repertoire was preserved, probably due to the complementary roles of temporal and parietal poles, and the distributed nature of socioemotional processing (Feinstein, 2013).

Standard group approximations to brain function are crucial the characterize the average brain and their neurocognitive functions. However, classical cognitive neuroscience views about neurocognition can be challenged by unusual individual cases. Alongside other lesion-based approaches, frontal compromise offers an informative model of development, resilience, and plasticity (Payne and Lomber, 2001). Comparably unexpected evidence has been offered in previous reports, including those of a man who led a completely normal life although he lacked 75% of his brain (Feuillet et al., 2007), a woman with highly preserved motor function despite primary cerebellar agenesis (Yu et al., 2015), another woman with multiple preserved functions even after two subsequent stroke affecting massive regions of her brain (García et al., 2016), patients who were able to restore their language skills after left hemispherotomy (e.g., Hertz-Pannier et al., 2002), or hydranencephaly patients with almost not cortices and preserved functions of consciousness, emotion, and basic sensorimotor abilities (Merker, 2007). In fact, these previous cases of preserved functions despite damage to critical regions have provided important insights into cognitive function and challenge current neurocognitive models and conceptions of brain organization and plasticity. Moreover, cases of structural underdevelopment, such as the present one, provide a powerful model revealing the self-sufficiency of specific neurocognitive mechanisms despite the absence of frontal structures. For instance, a predominantly behavioral profile (marked impulsive behavior, deficits of self-organization, and abstract reasoning) with partial preservation of other domains (e.g., memory, spatial skills, language) has been described in early stages frontal injuries (Price et al., 1990). However, this is the first report of the multidimensional impact of massive frontal-lobe underdevelopment with preservation of temporo-posterior structures. Thus, our case provides important insights regarding which critical functions resist developmental changes of the frontal lobes.

We are unable to definitively indicate whether frontal regions were displaced, or re-represented in other regions due to early neuroplastic changes or compensatory mechanisms. Frontal lobe affectation may have caused retrograde degeneration of the thalamus and transsynaptic degeneration of the cerebellum, as well as atrophy in the cerebral peduncles and diaschisis in the cerebellum. Given that these features were only partially present (Supplementary Fig. S1), the patient may still have the equivalent of some frontal functions but in a different location, triggered by neurodevelopmental changes. The partial anatomical preservation of these regions as well as the functional/structural network reorganization evidenced in the fMRI and DTI results suggests an early plastic reorganization, as observed on other conditions (Sarnat and Flores-Sarnat, 2016) and motor disorders (Rowe and Siebner, 2012).

Our study features the first report of DTI in frontal massive developmental changes. We found that fronto parietal and fronto-amygdaline connections (as well as other frontal connections) were lost. Only a few ventral connections (resembling a reduced section of the prefrontotectal pathways critical for attention (Gaymard et al., 2003)) were identifiable. Missing frontal tracts (uncinate, anterior cingulum) and atypical interconnections of temporo-posterior pathways suggest plastic and/or adaptive neurodevelopmental changes. Moreover, our results also evidence that except in ventral regions, no structural connections were identifiable in the remaining portions of frontal regions (e.g., the remaining small structures above of the corpus callosum). To our knowledge, this is the first human evidence that massive absence of frontal tracts involves atypical reorganization of cortico-cortical and cortico-subcortical connections.

This is also the first functional connectivity analysis in massive and neurodevelopmental frontal reorganization. Single-case analysis of connectivity (Dubois and Adolphs, 2016; Garcia et al., 2017; Sedeno et al., 2014) is useful to track the re-organization of abnormal brain networks. In line with DTI results, the anterior part of the amygdala and mid-IPS connections were absent. Nevertheless, basic organization of some posterior networks (amygdala, DMN) does not require frontal lobe integrity in the presence of developmental changes. This was also and especially true for other resting-state complexes that do not require direct functional coupling with frontal structures (i.e., the visual network). Nevertheless, a network requiring a critical role of frontal regions, such as the fronto-IPS and FPN (Fox et al., 2005; Hampshire and Sharp, 2015), presented an aberrant pattern connections. Finally, functional connections in the remaining portions of the frontal regions were absent, confirming the absent network activity in these regions.

This is the first assessment of this unique patient. Given the continual interruptions caused by the patient's conduct and the ensuing delays, only the reported behavioral tasks and recordings could be successfully completed. Though very challenging, it would be interesting for future studies on this subject to include EEG recordings during wakefulness and sleep as well task-based fMRI paradigms that normally engage the frontal lobes.

We were unable to confirm the pathogenesis and molecular basis of this case. Most neurodevelopmental disorders with absent brain structures involve neurogenetic or early compromise during embryogenesis. Certainly, this is not a case of holoprosencephaly, as no fusion in medial structures were identified. Neither could it be attributed to hydranencephaly, given the large portions of preserved cortex and absent phenotypic manifestations. Detailed visual inspection of MRI did not reveal (subependymal or subcortical) heterotopies, signs of migration alteration, schizencephaly or lissencephaly (alteration in cortical lamination). Nevertheless, the absence of higher resolution images precluded a definitive evaluation of these abnormalities. Only the remaining parenchyma in frontal locations showed fewer grooves and convolutions (pachygyria). The absence of prenatal imaging and genetic or histological data prevented clear neurodevelopmental diagnosis. In light of the patient's familial antecedents and phenotypical presentation, GC's seems to be a heterogeneous condition. The developing brain is highly sensitive to hydrostatic pressure generated internally, within the ependymal cavities, or externally, within the subarachnoid (meningeal) compartments (Budday et al., 2015). Hypoplasia does not follow from a simple pathogenic process. A combination of alterations in neural stem cell proliferation, apoptosis, neuronal migration, neuritogenesis, and connectivity alterations can be observed in different CNS hypoplastic regions (Budday et al., 2015; Lyss et al., 1999). During development, meningeal and nervous tissues interact by means of mesenchymal-neuroepithelial interactions. In fact, meninges are organized adapting to CNS morphogenesis (O'Rahilly and Müller, 2007). MRI images of the patient show the falx cerebri (sickle of the brain) correctly developed close to frontal and caudal parietal regions (Supplementary Fig. S2). This suggests normal development of the frontal lobes to a certain degree, at least until week 19. Though small, the majority of the sulci and gyri, as well as the corpus callosum, can be recognized in the images (Supplementary Fig. S3), further suggesting quasi-normal brain development up to week 30 (Bayer and Altman, 2006). The hypoplasic frontal lobes are displaced against the base of the skull and the frontal horns of the lateral ventricles are collapsed. Thus, the most plausible interpretation seems to be a prenatal intrauterine cyst filled with CFS during embryogenesis, which induced a secondary hypoplasia (dysplastic and underdeveloped) of the frontal lobe (for further considerations, see Supplementary discussion, Section 4).

## 5 Conclusion

Massive insults of the frontal lobes in early developmental stages can prove devastating for neurocognitive functions. This case demonstrates that even in the almost complete absence of frontal lobes, basic sensory, somatosensory, motor, emotional, and cognitive functions can be partially preserved. Conversely, critical frontal functions indexing domain-general skills (executive control and abstraction) were systematically affected. This profile of preserved and affected domains was supported by the specific pattern of brain structural and functional connections. Thus, even in the presence of functional compensation and neurodevelopmental plasticity, the frontal lobes seem critical for complex actions and thoughts demanding attention, abstraction, and control. Exceptional single cases like this one provide a challenge for current frameworks cutting across clinical science and current neuroscientific theories.

The following are the supplementary data related to this article.

Supplementary Video 1 Imitation, compliance with instructions, pretend play. Supplementary Video 1

Supplementary Video 2 Language comprehension, basic context-appropriate simulation. Supplementary Video 2

Supplementary Video 3 Finger-to-nose coordination, appropriate playful mood. Supplementary Video 3

Supplementary Video 4 Motor coordination upon demand, spontaneous pretend play with symbolic content. Supplementary Video 4

Supplementary Video 5 Partially preserved manual praxias. Supplementary Video 5

Supplementary Video 6 Partially preserved oro-facial praxias. Supplementary Video 6

Supplementary Video 7 Preserved simple praxias (tooth-brushing, hair brushing, waving), adequate verbal interaction. Supplementary Video 7

Supplementary Video 8 Receptive vocabulary, body-part recognition, basic object affordance recognition. Supplementary Video 8

Supplementary Video 9 Language-comprehension, self-other distinction, reality-fantasy discrimination, impaired abstraction capacity (addition skills). Supplementary Video 9

Supplementary Video 10 Pretend play (simulating unavailable communicative skills). Supplementary Video 10

Supplementary Video 11 MRI results. Supplementary Video 11

Supplementary material Image 1
