# Supplementary material for: Early bilateral and massive compromise of the frontal lobes
Source: Neuroimage Clin. 2018 Feb 27;18:543–52. doi: 10.1016/j.nicl.2018.02.026 (PMC5964834; doi:10.1016/j.nicl.2018.02.026)
Supplement: Supplementary file 12 — Supplementary material [file mmc12.docx]

**Early bilateral and massive compromise of the frontal lobes**

**Supplementary data**

**1. Neuropsychological assessment**

1.1. Attention

1.1.1 Visual attention

1.1.2. Auditory attention

1.2. Memory encoding

1.3. Language

1.4. Praxis

1.5. Emotional processing

**2. Results: Antecedents, neurological and neuropsychological outcomes**

2.1. Neurological examination

2.2. Neuropsychological assessment and evaluation

**3. Supplementary Figures**

3.1. Supplementary Figure S1. Additional visualization of brain structures.

3.2. Sup. Fig.S2. MRI sections illustrating anterior (frontal, A and B) and posterior (parietal, C and D) falx cerebri.

3.3. Sup. Fig. S3. MRI sections showing positions of principal sulci and gyri.

**4. Supplementary discussion**

**5. Supplementary videos**

5.1. Video 1. Imitation, compliance with instructions, pretend play

5.2. Video 2. Language comprehension, basic context-appropriate simulation

5.3. Video 3. Finger-to-nose coordination, appropriate playful mood

5.4. Video 4. Motor coordination upon demand, spontaneous pretend play with symbolic content

5.5. Video 5. Partially preserved manual praxias

5.6. Video 6. Partially preserved oro-facial praxias

5.7. Video 7. Preserved simple praxias (tooth-brushing, hair brushing, waving), adequate verbal interaction

5.8. Video 8. Receptive vocabulary, body-part recognition, basic object affordance recognition

5.9. Video 9. Language-comprehension, self-other distinction, reality-fantasy discrimination, impaired abstraction capacity (addition skills)

5.10. Video 10. Pretend play (simulating unavailable communicative skills)

5.11. Video 11. MRI results

**6. References**

**1. Neuropsychological assessment**

We implemented a two stage approach. First we used a systematized battery of neuropsychological functions (Bertoux et al., 2012; Bertoux et al., 2014; Rosselli Cock et al., 2004) including executive functions, attention, memory encoding, language, praxis and emotional processing (see Supplementary data, section 1, for detailed description of these tasks). Second, given that the patient refused to complete some specific subtests, insights into these domains were gained through a qualitative clinical interview following the recommendations for qualitative assessment frontal disorders (Mesulam, 2000). Some of these interactions were recorded and edited in short videos (see Supplementary videos).

**1.1. Attention**

**1.1.1 Visual attention**

Drawing cancellation task: The child was shown speciﬁc targets (44 big rabbits) and instructed to cross them out among an array of rabbits of different sizes, working row by row from left to right under time constraints. The total score was given by the number of hits (corrected for errors of commission). The maximum total score in this task is 44.

Letter cancellation task: The child was instructed to ﬁnd two matching letters among a line of letters, under time constraints. The total score was given by the number of hits. The maximum total score is 44.

**1.1.2. Auditory attention**

Digits Forward: The examinee had to repeat digits spoken by the examiner (span length is three to nine). The maximum total score is 8.

Digits Backward: The examinee had to orally reverse digits spoken by the examiner (span length is three to nine). The maximum total score is 8.

**1.2. Memory encoding**

Verbal memory Coding: The task involved four learning trials of 9 words each. The word list included three semantic categories: animals, fruits, and body parts. The maximum total score is 36.

Visual memory coding: The task involved four learning trials comprising 9 geometrical figures each. The child was instructed to remember one stimulus, and then retrace the ﬁgures from memory. The maximum total score is 36.

30-minute delayed recall

a) Verbal Recall: This task required free recall, cued recall, and recognition of the word list and free recall of a previously presented short story. The maximum total score is: 9, 9, and 18, respectively.

b) Visual Recall: This task required free recall, cued recall, and recognition of the geometrical figures presented previously. The maximum total score is: 9, 9, and 18, respectively.

**1.3. Language**

a. Expressive skills

Syllables repetition: The child was asked to repeat 8 syllables. The maximum total score is 8.

Word repetition: The child was instructed to repeat words in series of increasing length, to a maximum of 8 words. The maximum total score is 8.

Non-word repetition: non-words were aurally presented to the child, who then pronounced each stimulus. The maximum total score is 8.

Sentence repetition: This task involved novel sentences that cannot be easily recalled based on prior knowledge or by ﬁlling in missing words based on logic. The maximum total score is 8.

b. Comprehension

Picture naming: The child was required to name pictures of objects. The maximum total score is 15.

Oral commands: The child was required to point to the correct picture in response to commands of increasing syntactic complexity. The maximum total score is 15.

Discourse comprehension: This subtest taps auditory comprehension of spoken sentences. As such, it requires an understanding of grammar and semantic relationships independent of vocabulary level. The maximum total score is 10.

**1.4. Praxis**

Complex figure copying: This test requires copying and immediate free recall of a complex Rey figure-type design; the scoring system includes an overall summary score (based on presence, accuracy, and placement of elements) as well an evaluation of qualitative features: fragmentation, planning, and organization. The maximum total score is 12.

Visual perception: This domain was assessed with the line orientation (identifying lines within a model) and coordinate-location (drawing a route using visual directions on a 6 x 6 array of one-cm squares) tasks.

Superimposed figures: Three sheets with superimposed drawings were presented. The first sheet had six figures; the second, five; and the third, five. A point was given for each recognition. The maximum total score is 16.

**1.5. Emotional processing**

Emotion recognition. The emotion recognition task was obtained from the Social Cognition and Emotional Assessment battery (Bertoux et al., 2012; Bertoux et al., 2014). This task provides a composite and individual scores from the facial emotion recognition test (from Ekman’s pictures; including fear, disgust, anger, surprise, sadness, and happiness; scored from 0 to 15). The patient was asked to identify which emotion is being expressed in each base. Full details and explanations are available elsewhere (Bertoux et al., 2012; Bertoux et al., 2014).

**2. Results: Antecedents, neurological and neuropsychological outcomes**

**2.1. Neurological examination**

During neurological examination, GC was conscious, awake, and partially attentive. She was able to describe sensory and affective experiences, and reacted to environmental events with congruent emotional and cognitive responses (e.g., pleasure, tiredness, playfulness, anger, and basic symbolization; see Supplementary Videos 1-2). She made explicit self-other distinctions (see Supplementary Video 9) and recounted personal experiences. Her speech was fluent and acceptably intelligible, with preserved repetition, comprehension, and naming. GC also exhibited a vast range of social behaviors (e.g., smiling, intersubjective involvement, imitation, active questioning; see Supplementary Videos 1-2, 7, 9-10). However, she sometimes acted impulsively and was easily distracted.

She exhibited motor perseverance, but muscle bulk and tone were normal. Strength was full bilaterally. Bilateral mirror movements were observed in hands and feet, as well as difficulties with motor planning (motor sequences). Posture was normal. Despite an unsteady gait and difficulties to stop walking, tandem gait and gait speed were preserved and no focal paresis was observed. Rapid alternating movements and fine finger movements were affected. There was partial dysmetria on finger-to-nose (see Supplementary Video3) and heel-knee-shin. Regarding ocular movements, normal saccades and normal threat reflexes were observed. Her pupils were isochoric, and she gave no signs of strabismus, nystagmus, or abnormal ocular motility. Suck, snout, and palmomental reflexes were present, indicative of frontal motor compromise. The cranial nerves (from II-XII) were preserved. Reflexes were 2+ and symmetric at the biceps, triceps, knees, and ankles. She also presented plantar responses (bilateral Babinski reflex).

Evaluation of the sensory system revealed no gross abnormalities in the fingers, toes or other extremities. Light touch, pinprick, position sense, and vibration sense were intact in fingers and toes. Her facial features gave no indications of any kind of holoprosencephaly. Neither were there any signs of cerebral palsy, hypothalamic dysregulation, poor gastric emptying, temperature regulation, central diabetes, or anterior pituitary dysfunction.

**2.2. Neuropsychological evaluation**

GC’s demeanor was adequate. She was cooperative. She gave no signs of apathy or negativity, but her behaviors were disruptive and perseverative, requiring continuous external control. She was also disinhibited, with poor emotional and cognitive regulation. Here we report the most affected and preserved domains separately.

*Most affected domains: executive control and abstraction*

Poor control and regulation of behaviors were the hallmark of GC’s deficits (frontal disinhibition syndrome). No signs of frontal abulic syndrome were evident. Although not observed during the interview, her mother described systematic disinhibited and inappropriate behavior at home and school. These behavioral impairments emerge in real-life situations (when behavior is under minimal external control) but not in the structured interviews. During the interview, she could not perform activities requiring sustained attention (with pronounced persistence and perseveration), and benefited from external orientation (e.g., gestures) from an adult. Inhibitory control was markedly compromised. Her verbal working memory was only partially spared (forward and backward digit span), but she had poor symbol-manipulation skills. Her performance was characterized by intrusions, contaminations, and perseverations. Frontal release signs (primitive suck, snout, and palmomental reflexes) were indicative of large structural affectation compromising premotor areas.

Abstract thought was systematically impaired. GC was not able to perform basic additions or subtractions, neither spontaneously nor upon demand (Video 9). Reading, writing, mathematical, and especially graphic stimulus manipulation were under-automatized. She was unable to provide explicit abstract definitions or concepts.

*Partially preserved domains: language, spatial orientation, memory, and socioemotional behavior*

GC was spontaneously communicative, with preserved naming skills and partially spared spontaneous production and repetition of single words and short sentences (Videos 1, 2, 7-9). Comprehension was preserved, as the patient was able to follow conversation, ask yes/no questions, and point to appropriate objects. She established eye contact with her interlocutors, and expressed context-sensitive ideas and emotions. Verbal knowledge of objects and body parts was partially spared (Video 8). Basic pragmatic skills (attitude, affect, prosody) were preserved. Articulatory difficulties (kinetic melody, articulema), paraphasic deficits (phoneme omissions and substitutions), and delayed morphosyntactic skills were observed. She was unable to read or write.

Regarding memory, the patient was able to narrate autobiographical events relying on declarative (explicit) memory. No signs of retrograde and anterograde amnesia, prosopagnosia, object agnosia, or anomia were observed. Regarding gnosias, GC recognized shapes, sizes, colors, body images, and sounds in the environment. However, she was impaired in recognizing complex objects.

As regards spatial orientation, extrapersonal space attention was partially impaired, but no evidence of hemispatial neglect, simultanagnosia, and object finding deficits was identified. Partial disruption of self-centered and object-centered coordination was observed for obstacle avoidance, although dressing praxias preserved. Simple and orofacial, oculomotor, and optic praxias were preserved (Video 6). However, despite basic motor preservation, complete construction, kinetic, and (partial) ideomotor apraxia were identified (Videos 5-7). Poor dissociative fluency for graphomotor actions was also observed.

Finally, during social interactions, GC expressed happiness, anger, fear, and surprise. She was able to simulate emotional face- and body-language expressions (Videos 1, 2, 9-10). GC also exhibited a vast range of social behaviors (e.g., smiling, intersubjective involvement, imitation, active questioning; Videos 1, 2, 7, 9-10). Furthermore, she had above-chance recognition of basic emotional states critical for social interactions, such as happiness, disgust, and anger. Other emotional states (fear, surprise, neutral) were not well identified. She spontaneously engaged in fantasy-based playful behavior and spontaneous symbolic play (Video 1, 2, 4, 9-10). She also showed basic skills to infer others’ internal states, although she refused to undergo formal theory-of-mind assessment.

**3. Supplementary Figures**


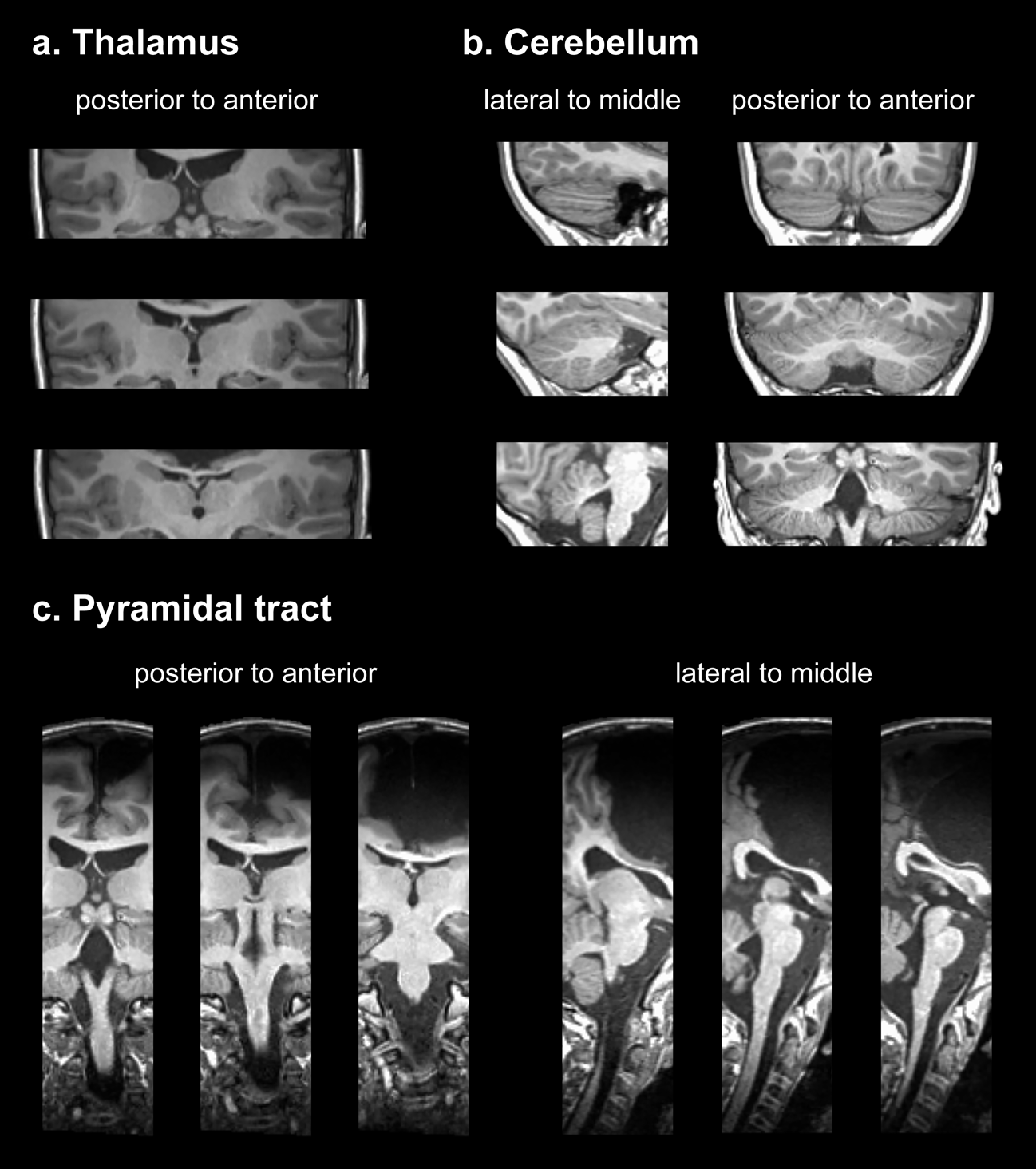


**Supplementary Figure S1. Additional visualization of brain structures.** Original T1 sequence showing coronal view of the thalamus, including the medial dorsal nucleus, VA and VL (a); sagittal and coronal view of the cerebellum (b) and pyramidal tract (c). Note that there were not clear sings of potential retrograde degeneration in the thalamus, and the cerebellum did not shown signs of diaschisis or similar alterations. All images are showed in neurological orientation.


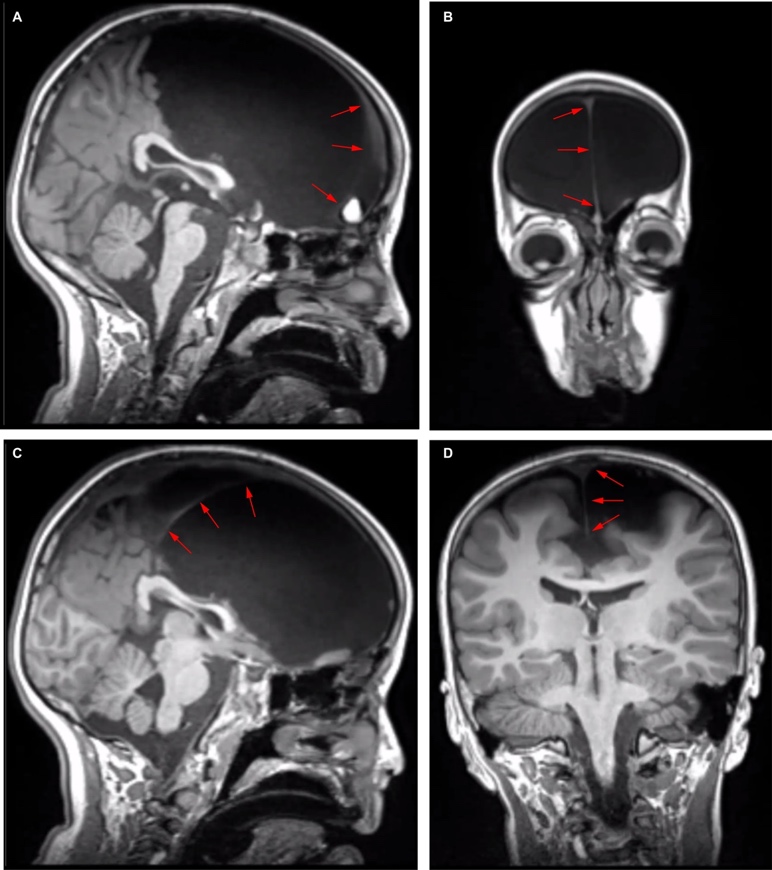


**Supplementary Figure. S2. MRI sections illustrating anterior (frontal, A and B) and posterior (parietal, C and D) falx cerebri.** The falx cerebri is present in areas lacking brain parenchyma. Images show the presence of a large extracerebral cavity (external to the CNS) that does not correspond to ventriclular dilation. This large external cavity seems to partially compress the brain, especially in the preserved sections of the frontal lobes.


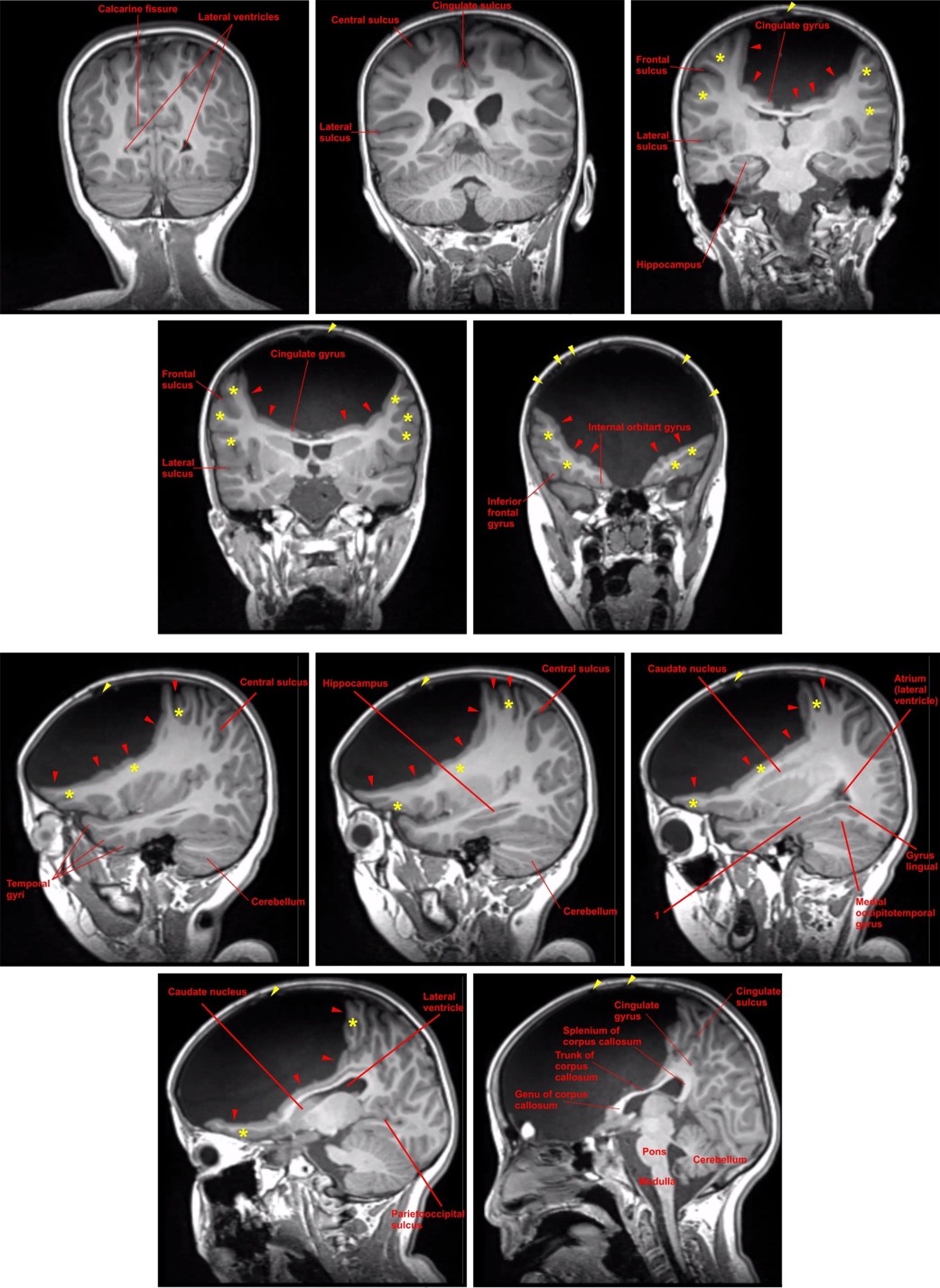


**Supplementary Figure S3. MRI sections showing positions of principal sulci and gyri.** Coronal sections show medial-lateral displacement and compression of the frontal lobes. Parasagittal sections show apparent cephalic-caudal displacements. Red arrowheads indicate zones of cortical hypoplasia (small gyri or absence of gyri, thinner gray matter). Yellow asterisks indicate apparent collapsed and hypoplasic section of frontal cortex. Yellow arrowheads indicate discontinuities in meninges.

**4. Supplementary Discussion**

Given the patient’s specific brain features, the potential differential diagnosis includes interhemispheric fissure arachnoid cyst, neuroglial cysts, and epidermoid cyst located in the interhemispheric fissure. Brain regions adjacent to the cyst show reduced overall cortical surface and cortical thickness. Little is known about the influence of cysts on the organization of adjacent cortices. In some cases, the hemispheric asymmetry of classical language regions remains unchanged (Hund-Georgiadis et al., 2002). However, evidence from somatomotor functional MRI shows significant activation changes in cortical and cerebellar areas in an asymptomatic patient with a congenital cyst, demonstrating an alternative organization of the central motor system with a preservation of basic neurological function (Alkadhi et al., 2003). Early development of these pathologies may explain adaptive and plastic mechanisms that functionally compensate for the reduction of brain volume and the compression of the parenchyma. The present study shows that structural alterations (hypoplasia, underdevelopment, compression) are accompanied of drastic changes in the structural and functional connections among the basal and subcortical regions with dorsal frontal areas, evidencing plastic reorganization of other regions.

**5. Supplementary videos**

5.1. Video 1. Imitation, compliance with instructions, pretend play

5.2. Video 2. Language comprehension, basic context-appropriate simulation

5.3. Video 3. Finger-to-nose coordination, appropriate playful mood

5.4. Video 4. Motor coordination upon demand, spontaneous pretend play with symbolic content

5.5. Video 5. Partially preserved manual praxias

5.6. Video 6. Partially preserved oro-facial praxias

5.7. Video 7. Preserved simple praxias (tooth-brushing, hair brushing, waving), adequate verbal interaction

5.8. Video 8. Receptive vocabulary, body-part recognition, basic object affordance recognition

5.9. Video 9. Language-comprehension, self-other distinction, reality-fantasy discrimination, impaired abstraction capacity (addition skills)

5.10. Video 10. Pretend play (simulating unavailable communicative skills)

5.11. Video 11. MRI results

**6. References**

Alkadhi, H., Crelier, G.R., Imhof, H.G., Kollias, S.S., 2003. Somatomotor functional MRI in a large congenital arachnoid cyst. Neuroradiology 45, 153-156.

Bertoux, M., Delavest, M., de Souza, L.C., Funkiewiez, A., Lepine, J.P., Fossati, P., Dubois, B., Sarazin, M., 2012. Social Cognition and Emotional Assessment differentiates frontotemporal dementia from depression. J Neurol Neurosurg Psychiatry 83, 411-416.

Bertoux, M., Volle, E., de Souza, L.C., Funkiewiez, A., Dubois, B., Habert, M.O., 2014. Neural correlates of the mini-SEA (Social cognition and Emotional Assessment) in behavioral variant frontotemporal dementia. Brain Imaging Behav 8, 1-6.

Hund-Georgiadis, M., Yves Von Cramon, D., Kruggel, F., Preul, C., 2002. Do quiescent arachnoid cysts alter CNS functional organization?: A fMRI and morphometric study. Neurology 59, 1935-1939.

Mesulam, M.M., 2000. Principles of Behavioral and Cognitive Neurology. Oxford.

Rosselli Cock, M., Matute Villasenor, E., Ardila Ardila, A., Botero Gomez, V.E., Tangarife Salazar, G.A., Echevarria Pulido, S.E., Arbelaez Giraldo, C., Mejia Quintero, M., Mendez Losado, L.C., Villa Hurtado, P.C., Ocampo Agudelo, P., 2004. Neuropsychological Assessment of Children: a test battery for children between 5 and 16 years of age. Neurology 38, 720-731.
